# Nonlinear Flow Sensor Calibration with an Accurate Syringe

**DOI:** 10.3390/s18072163

**Published:** 2018-07-05

**Authors:** Paolo Jose Cesare Biselli, Raquel Siqueira Nóbrega, Francisco Garcia Soriano

**Affiliations:** 1 Intensive Care Unit, University Hospital, University of Sao Paulo, Av. Prof Lineu Prestes, 2565, Butantã, São Paulo, SP 05508-000, Brazil; raquelsnobrega@hu.usp.br (R.S.N.); gsoriano@usp.br (F.G.S.); 2 Clinical Emergencies, Medical Clinical Department, University of Sao Paulo, Av. Dr. Arnaldo, 455, sala 3132, São Paulo, SP 01246 903, Brazil

**Keywords:** flow sensor, calibration, mechanical ventilation, nonlinear

## Abstract

Flow sensors are required for monitoring patients on mechanical ventilation and in respiratory research. Proper calibration is important for ensuring accuracy and can be done with a precision syringe. This procedure, however, becomes complex for nonlinear flow sensors, which are commonly used. The objective of the present work was to develop an algorithm to allow the calibration of nonlinear flow sensors using an accurate syringe. We first noticed that a power law equation could properly fit the pressure-flow relationship of nonlinear flow sensors. We then developed a software code to estimate the parameters for this equation using a 3 L syringe (calibration syringe). Finally, we tested the performance of a calibrated flow sensor using a different 3 L syringe (testing syringe) and a commercially available spirometer. After calibration, the sensor had a bias ranging from −1.7% to 3.0% and precision from 0.012 L to 0.039 L for volumes measured with the 3 L testing syringe. Calibrated sensor performance was at least as good as the commercial sensor. This calibration procedure can be done at the bedside for both clinical and research purposes, therefore improving the accuracy of nonlinear flow sensors.

## 1. Introduction

Management of patients in mechanical ventilation requires proper measurement of respiratory pressure, flow, and volume. Several statements in this field recommend maximal values for inspiratory pressure and delivered tidal volumes [1,2,3,4]. Flow and pressure measurements can be used to guide clinical care and are widely used in respiratory research. In all applications, sensor calibration is vital for obtaining reliable measurements.

Manufacturers of mechanical ventilators have incorporated some calibration procedures in their devices. However, tidal volumes set on ventilators often differ from values measured with calibrated flow sensors [5,6,7], particularly in children and when flows are not measured at airway opening [8,9]. In a multicentric analysis of different mechanical ventilators, flow-dependent parameters were the most error-prone measurements [10]. For the same set tidal volume, true delivered tidal volumes can change with variation in resistance and compliance [11]. Therefore, improvements in flow measurement technique is important for delivering better care to patients [12].

In procedures requiring accurate flows, as in spirometry and respiratory research, calibration is usually performed daily [13]. Frequent calibration using flows within the range of interest can ensure better accuracy of recorded data, particularly for nonlinear sensors [14]. A calibration procedure requires generating accurate standards of the parameter of interest to which the sensor reading will be compared. After several measurements, sensor reading is updated to match the generated standards, thereby completing the calibration. While one can easily obtain pressure standards, accurate flow standards are often difficult to produce. Therefore, precision syringes have been used as standards for flow sensor calibration [13,15].

In the syringe calibration method, the total content of an accurate syringe is emptied through the flow sensor at variable speeds. The recorded signal is time-integrated and set to equate the volume of the syringe. This is an easy and inexpensive method for linear flow sensor calibration and eliminates the need for generating accurate flow standards. Several flow sensors in use are disposable and cheap but often have a nonlinear behavior, which increases the complexity for the syringe calibration method. Tang and co-workers have acknowledged the nonlinear relationship of flow sensors and described a polynomial method for calibrating these sensors [15].

As syringe-integral method is very useful for bedside calibration, we developed an alternative algorithm to allow the use of this calibration procedure with a nonlinear flow sensor. After finding calibration parameters, we analyzed the performance of the nonlinear calibrated flow sensor using a different accurate syringe and a commercial spirometer.

## 2. Materials and Methods 

### 2.1. Equipment

We used a nonlinear flow sensor (VarFlex, Viasys Healthcare, Palm Springs, CA, USA) for the experiments. This sensor is used in Bicore Monitoring system (Pulmonary Monitor CP 100, Bicore Monitoring System, Irvine, CA, USA). Similar versions of the sensor are in use in several mechanical ventilators, such as Hamilton (Hamilton Medical AG, Bonaduz, Switzerland), VELA (Vyaire Medical, Mettawa, IL, USA), and Intermed ventilators (CareFusion, São Paulo, SP, Brazil).

We connected the flow sensor to a preamplified differential pressure sensor (HSC Series pressure range 0–10 cm H_2_O, Honneywell, Columbus, OH, USA), attached to an analog–digital converter (NI USB-6008, National Instruments, Austin, TX, USA). We recorded signals in LabView (LabView 2013, National Instruments, Austin, TX, USA). No subjects were used in the process. We used an accurate 3 L syringe (Alpharad, Santo André, SP, Brazil) for developing the calibration procedure (calibration syringe).

### 2.2. Describing Flow-Pressure Relationship in Our Nonlinear Flow Sensor

The differential pressure sensor has a linear pressure-voltage response at the nominal range. Therefore, flow-voltage nonlinearity can be attributed to the nonlinear relationship between flow and pressure. 

**Figure 1:**
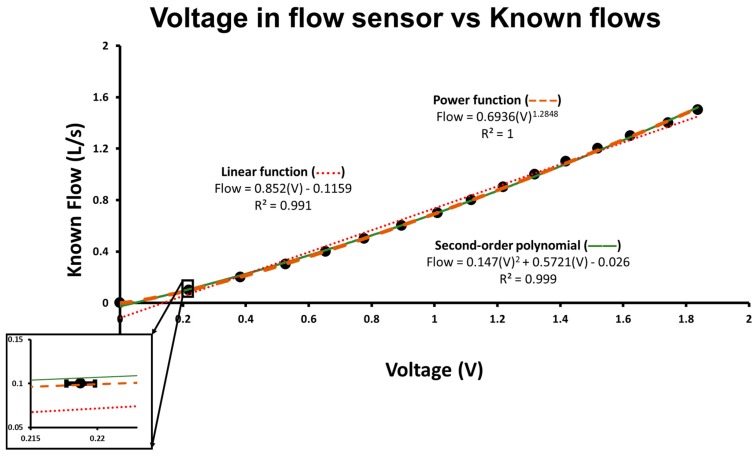
Known flows versus non-calibrated flow sensor (in volts). The graph represents the non-calibrated recordings provided by the flow sensor at different known flows that were generated by a mechanical ventilator (Evita XL). The ventilator was set to volume control with constant flow and connected to the flow sensor. The flow was progressively increased, and we recorded values (in Volts) provided by the non-calibrated flow sensor. Each point in the graph is the average of 15 measurements at each flow step. Error bars represent standard error of mean but are too small to be viewed in the main graph. A subset of the graph is shown in detail (for the second flow point) to allow the visualization of the size of standard error of mean. Regression lines for linear function (dots), power function (traces), and second order polynomial (line) with their respective equations are shown. Compared to the linear function, the power function improves the fitting and accounts for the nonlinear pressure-flow relationship.

First, we analyzed flow-pressure relationship with a mechanical ventilator (Evita XL, Dräger, Lübeck, Germany). We set the ventilator to volume control and delivered incremental flow rates to the test flow sensor. We then plotted nominal flows versus the voltage recorded using the flow sensor. At each flow step, we measured voltage in the flow sensor in 15 different trials and calculated the average and the standard error of mean. The flow-voltage (and therefore flow-pressure) relationship of the flow sensor could then be appreciated (Figure 1). A power equation (Equation (1)) could adequately fit the nonlinear voltage-flow relationship and was therefore selected for the algorithm construction.

Flow = A*(voltage-**c**)**^b^**, if voltage ≥ c Flow = −A*(**c**-voltage)**^b^**, if voltage < c (1)

where ‘A’ and ‘b’ are calibration parameters and ‘c’ is baseline voltage at zero flow. Nominal flows generated by the ventilator were only used to observe the nonlinear relationship and to explore which calibration function would be used. We did not use those flows for the subsequent calibration procedure.

### 2.3. Construction of Calibration Algorithm

During the calibration procedure, the full content of a 3 L calibration syringe was emptied through the flow sensor in 8 trials at different speeds, producing flows within the range of interest (between 0.1 and 1.6 L/s). Operators did not need to generate constant flows and were able to observe the non-calibrated flows in real time. They could adapt the pace of the stroke to obtain values in the desired range. If recorded flows were not within the range of interest, the procedure was repeated.

As the sensor is bidirectional but calibration parameters may change between different directions, we undertook 8 trials in each direction (inspiration, positive flows; and expiration, negative flows). The collected data for inspiration and expiration were analyzed separately.

**Figure 2:**
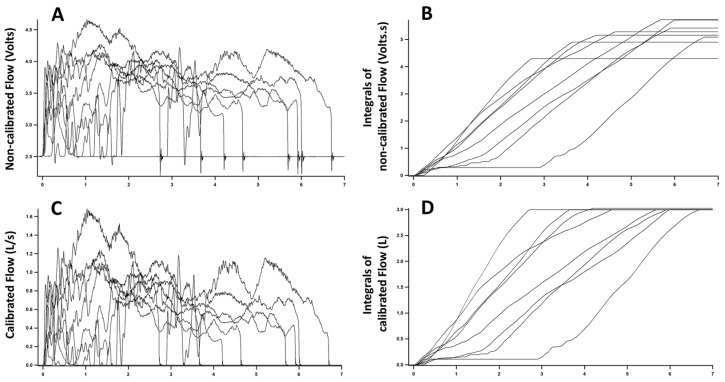
Calibration trials and procedures. The graphs represent recordings of 8 trials with a 3 L syringe (calibration syringe) emptied through the flow sensor at different speeds. Signals recorded by the flow sensor prior to calibration are displayed in (**A**) and were integrated over time (**B**). Although all trials were done with the same syringe, the integrals have different final values, underscoring the nonlinearity of the flow sensor. A software algorithm was used to find parameters for a power function minimizing the difference between integrals [Flow = A*(voltage^b^)]. (**C**) Shows flow signals after application of calibration parameters. (**D**) Shows the integrals of calibrated flow signals. Note that after calibration parameters were used, integrals are equal among each other and equal to the volume of the syringe (compare to **B**).

Recorded signals were transferred to a data analysis software (IgorPro 6, WaveMetrics, Portland, OR, USA). We measured parameter ‘c’ as the average of a segment with no flow and subtracted ‘c’ from recorded signals. We inverted negative values to avoid exponentiation of negative numbers and time-integrated signals. As the sensor is nonlinear, integrals obtained with different flows did not show a constant value despite being produced with the same syringe (Figure 2A,B). We recalculated the integrals to search for an optimal power function that would minimize the difference in the integrals obtained in the various trials. For this purpose, we coded a loop in IgorPro6 software that would apply different exponents to the measured flows, repeat the time-integration, and search for the exponent producing the smallest difference among the integrals (Figure 2C,D). Once the exponent ‘b’ was found (Equation (1)), parameter ‘A’ was determined to equate the value of the integral to the volume of the calibrated syringe (3 L). As the time scale used was seconds, this procedure calibrated flow to L/s.

We repeated the procedure for the other direction of the sensor. In general, calibration parameters were slightly different between the two directions (positive and negative flows). The approximate time required for the whole calibration was 15 min but further automation can expedite this process.

### 2.4. Evaluating the Calibration Parameters

The ‘A’ and ‘b’ parameters for each of the directions were then inserted back in the LabView system (LabView 2013, National Instruments, Austin, TX, USA) to transform the recorded signals from voltage to calibrated flow (L/s). Parameter ‘c’ was recalculated as baseline voltage with no flow and was subtracted from voltages that were subsequently measured. Then, parameters ‘A’ and ‘b’ for each specific direction (positive or negative) were used according to Equation (1). If voltages were negative, we inverted values for exponentiation and reinverted the result after that. We tested the calibration parameters with a different 3 L syringe (testing syringe) (Welch Allyn, Skaneateles Falls, NY, USA).

We compared the calibrated flow sensor to a commercially available spirometer (microQuark, Cosmed, Rome, Italy). We used the 3 L calibration syringe to deliver a series of 8 stroke volumes simultaneously to both the calibrated flow sensor and Cosmed sensor. We displayed flows measured with both devices over time and Bland-Altman plot comparing the measurements [16]. Additionally, we used both syringes (calibration and testing syringe) to generate a series of 13 stroke volumes to both the calibrated flow sensor and Cosmed sensor. Values in the Cosmed sensor were converted to ambient temperature, pressure, and humidity. We compared volumes read to nominal values of the syringes (3 L). Precision (standard deviation of the several trials) and bias (percent deviation from the standard value, which is a measure of trueness) were calculated for both flow sensors according to ISO 5725 definition [17] and recommendations for sensor performance analysis [14].

Finally, we tested the ability of the proposed algorithm to obtain adequate calibration parameters with three additional flow sensors (Versamed, Kadima, Israel; Intermed/CareFusion, Sao Paulo, SP, Brazil; Novametrix Medical System, Wallingford, CT, USA). Moreover, we compared the performance of the calibrated flow sensor to the expiratory flow sensor of Evita XL mechanical ventilator while ventilating a testing balloon.

## 3. Results

The nonlinear flow-pressure relationship can be appreciated in Figure 1. Using flows generated by a mechanical ventilator (Evita XL), we built a flow-voltage curve revealing the nonlinearity between flow and pressure within the range of interest. A linear function can provide a reasonable calibration, but the slight deviation from the regression line prevents an adequate calibration using the syringe method (Figure 2B). A power law equation can account for the observed nonlinear relationship and was subsequently used in the development of the calibration algorithm. A second order polynomial, as previously described [15], could also adequately fit the curve but was not superior to the power function.

The nonlinearity of flow-pressure relationship is further detected in Figure 2. In Figure 2A, we observe the variable flow patterns generated with the 3 L syringe. However, despite the total volume being constant (the volume of the syringe), the integrals of non-calibrated signals differ from each other (Figure 2B). The nonlinear flow-pressure relationship causes the disparity in integrals of trials using different flow rates and prevents the straightforward use of the syringe calibration method.

After finding power function calibration parameters, flow signals are converted from V to L/s (Figure 2C). At this point, integrals of flow signals are equal to each other and to the volume of calibration syringe irrespective of the flow pattern used (Figure 2D).

**Figure 3:**
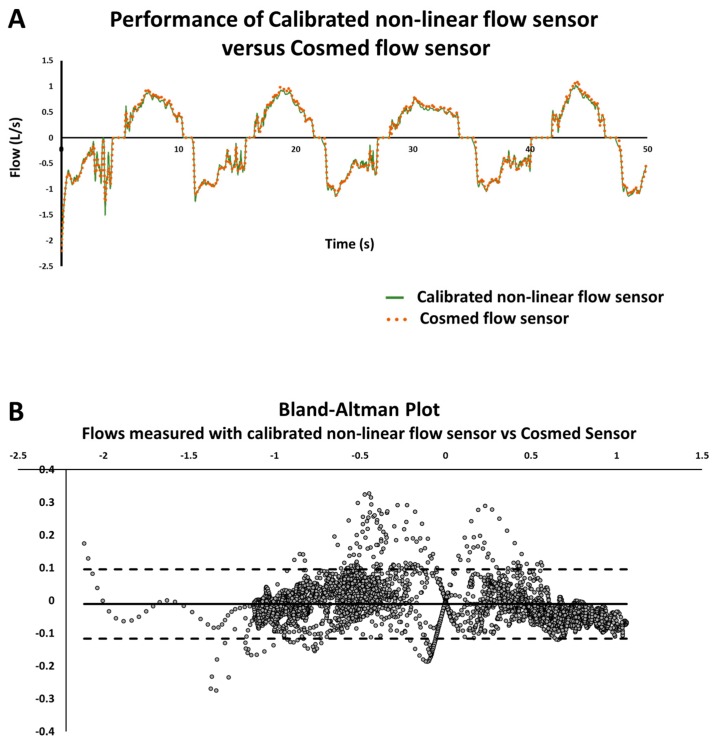
Comparison of calibrated nonlinear flow sensor with Cosmed flow sensor (converted to ambient conditions of pressure, temperature, and humidity). (**A**) Shows the performance of both flow sensors over time in a series of strokes generated with the 3 L calibration syringe. (**B**) Presents data recorded above displayed in a Bland–Altman plot. Each point represents the difference in flows measured with both sensors versus the average flow between both devices. The solid line represents bias, while the dashed lines represent limits of agreement (1.96 SD of differences from the bias).

Figure 3 shows the comparison between flows measured with nonlinear calibrated flow sensor and commercial flow sensor (converted to ambient conditions of pressure, temperature, and humidity). In Figure 3A, we observe the performance of both sensors over time and can notice the good agreement between the two. Figure 3B displays the Bland–Altman plot comparing flows measured with both flow sensors. Bias (solid line) is −0.011 L/s and limits of agreement (dashed lines) are −0.1169 L/s and 0.0949 L/s.

Table 1 displays the performance of the nonlinear calibrated flow sensor on at least 13 trials in each direction (positive: inspiratory; and negative: expiratory) with both the 3 L syringes (calibration syringe and testing syringe) after calibration parameters were used. Precision after calibration was between 0.012 L and 0.070 L, while bias ranged from −1.7% to 3.0%. The maximal error found in the trials was 4.5%. For comparison, we also display the performance of the Cosmed flow sensor using both syringes. The calibrated flow sensor performed at least as well as the commercial flow sensor.

The developed algorithm also performed well in the three additional nonlinear flow sensors tested (Novametrix, Intermed/CareFusion and Versamed). In all cases, we obtained adequate calibration parameters that could equate integrals measured with variable flow rates to the volume of the calibration syringe (data not shown). Tidal volumes read by Evita XL expiratory flow sensor were also compared to calibrated nonlinear flow sensor in a set of 39 trials while ventilating a balloon. The average difference between measurements was 2.1% and maximal error was 6.1%.

## 4. Discussion

In our study, we present an algorithm that allows the use of an accurate syringe to calibrate nonlinear flow sensors. We observed that a power function could adequately compensate for the sensor nonlinearity. A software-generated loop was used to find parameters for the power function and allow an adequate bedside calibration procedure.

There are several methods for measuring flow [14,18], but clinical and medical research applications widely use flow sensors based on pressure drop across a resistance [19]. Despite the importance of measuring flow, little importance is given to adequate calibration [20]. Several authors have observed significant lack of consistency between volumes set at mechanical ventilators and volumes actually delivered to patients [5,6,7,8,9]. Therefore, improvements in systems for flow measurement are required [12] and proper calibration can contribute to this goal.

Flow sensors can be particularly cumbersome to calibrate because generating accurate standard flows is difficult. A simple solution is the use of an accurate syringe [13,15]. In this method, the operator does not need to generate known flows but performs the calibration by time-integrating flow signal. This is a simple and reliable calibration procedure.

The syringe calibration method is adequate for linear flow sensors. However, many disposable flow sensors are nonlinear and even so-called linear flow sensors display some nonlinear relationship over the reading range of interest. The use of the syringe method for calibrating this type of sensor is challenging [15] and developing an algorithm to allow this calibration is useful for improving accuracy.

After generating a wide range of flows with a mechanical ventilator, we observed the nonlinearity of the flow sensor used and noted that a power law equation could solve the nonlinear voltage-flow relationship (Figure 1). In order to use the syringe calibration procedure, we designed an iterative algorithm to find the parameters for the power function.

Using this technique, we were able to calibrate the nonlinear sensor and determine parameters that would result in the correct syringe volume reading even when a wide range of flows (0.1 to 1.6 L/s) was used. For patients in mechanical ventilation, this range of flows is sufficient to represent the breathing pattern of the vast majority of adults. The calibration performance was validated with a different accurate volume syringe (3 L testing syringe). The flow sensor performed well in that test, with a precision around 0.03 L, bias ranging from −1.7% to 3.0%, and maximal error of 4.5%. This compares well with flow sensors in mechanical ventilators. The flow sensor in Evita XL mechanical ventilator has an accuracy of 8% of the measured value for both flow and tidal volume, as reported by the manufacturer.

Both second order polynomial [15] and power equation could be used to account for the nonlinearity of the flow sensor. We chose to evaluate the performance of a power equation because a simple iteration could be used to compute the parameters for the calibration equation. We did not compare the performance of both calibration procedures, but the curve fitting in the flow-voltage curve (Figure 1) showed the second order polynomial was not superior to the power equation in accounting for the nonlinear relationship.

We did not add correction factor for gas viscosity, humidity, and temperature. In a screen pneumotachograph, like the one used in this study, changes in air density, temperature, and humidity can alter the pressure-flow relationship [21]. Our algorithm was designed to compensate for the structural nonlinear pressure-flow relationship of flow sensors. During calibration with a syringe, there are no changes in humidity, temperature, or FiO_2_ that would justify additional adjustments. However, when applied to patients, correction to BTPS (Body Temperature and Pressure Saturated) should be added, and an additional correction factor for gas viscosity can be incorporated according to equations previously described [21,22,23]. Because pressure drop across the flow sensor is a function of gas viscosity for both turbulent and laminar flows [18], previously described correction factors for FiO_2_ and Heliox should also apply for this type of flow sensors.

## 5. Conclusions

In the present work, we describe an algorithm to enable the use of an accurate syringe to calibrate nonlinear flow sensors. The procedure is simple and can be done at the bedside, which will improve the quality of collected flow and volume data for both research and clinical applications.

## Figures and Tables

**Table 1:** Performance of the calibrated flow sensor with two 3 L syringes (calibration syringe and testing syringe) compared to a commercial spirometer (Cosmed). Values in both flow sensors are presented in ambient temperature, pressure, and humidity conditions.

	Trueness		Precision		Bias		Maximal Error (%)	
	(Average of Trials—L)		(Standard Deviation—L)		(% Change from True Value)			
***Flow sensor***	*Non-linear*	*Cosmed*	*Non-linear*	*Cosmed*	*Non-linear*	*Cosmed*	*Non-linear*	*Cosmed*
	*calibrated*		*calibrated*		*calibrated*		*calibrated*	
**3 L Calibration syringe**								
**Inspiration**	2.98	3.00	0.018	0.040	–0.6%	–0.2%	1.6%	3.1%
**Expiration**	3.03	3.05	0.070	0.064	1.0%	1.7%	4.5%	3.4%
**3 L Testing syringe**								
**Inspiration**	2.95	3.08	0.012	0.053	–1.7%	2.6%	2.2%	5.9%
**Expiration**	3.09	3.14	0.039	0.050	3.0%	4.7%	4.2%	7.8%
